# Using rapid-sequence MRI scans to compare ventricular and intracranial volumes in shunted and non-shunted children with myelomeningocele: a retrospective observational cohort study

**DOI:** 10.1007/s00381-026-07410-w

**Published:** 2026-07-24

**Authors:** Maya Parker, Oumou Kalsoum Mbacke, Divine Nwafor, Abhishek Kumar, Xue Feng, Ryan Kellogg, David Martin, Amanda Hendrix, Heather Spader

**Affiliations:** 1 Department of Neurosurgery, University of Virginia, 1240 Lee St, Charlottesville, VA 22902 USA; 2 Department of Biomedical Engineering, University of Virginia, Charlottesville, VA USA; 3 Claude Moore Health Science Library, University of Virginia School of Medicine, Charlottesville, VA USA

**Keywords:** Spina bifida, Hydrocephalus, Ventriculoperitoneal shunt, Ventricle-to-intracranial volume ratio

## Abstract

**Purpose:**

Hydrocephalus is a common complication of myelomeningocele (MMC), typically managed with a ventriculoperitoneal shunt (VPS). Despite clinical relevance, there remains a paucity of studies quantitatively comparing intracranial and ventricular volumes in shunted versus non-shunted MMC patients. This study aims to determine whether significant volumetric differences exist between these groups.

**Methods:**

Ninety-one MRIs from 19 MMC patients (13 shunted, 6 non-shunted), aged 0–3 years, were retrospectively analyzed. Volumes were obtained from rapid-sequence MRIs using automatic segmentation with manual correction. Outcomes included ventricular volume, intracranial volume, and ventricle-to-intracranial volume (ventricle/ICV) ratio. Analyses included nonparametric tests, mixed-effects models, bootstrap resampling, permutation testing, and robust regression.

**Results:**

At baseline, shunted patients exhibited larger intracranial volumes (644.3 vs 403.5 mL; *p* = 0.020) but not ventricular volumes (82.2 vs 34.2 mL; *p* = 0.072) compared to non-shunted patients. Although the ventricle-to-intracranial volume ratio was significantly higher in shunted patients under 50 days of age (*p* = 0.026), this difference was no longer significant after adjusting for head circumference and prematurity (*p* = 0.144). Sensitivity analyses confirmed differences in the neonatal period (*p* = 0.013), but not in infancy or toddlerhood. Effect-size analyses demonstrated a large difference within 50 days (Hedges’ *g* = 1.268) that diminished thereafter (*g* = 0.452).

**Conclusion:**

Shunted MMC patients demonstrated larger intracranial volumes and higher ventricle/ICV ratios in the neonatal period, but these differences diminished after 50 days. Early ventricular enlargement in this cohort’s shunted patients appears robust yet transient, with no sustained volumetric differences between shunted and non-shunted patients later in development.

## Introduction

Spina bifida is a congenital neural tube defect characterized by incomplete closure of the spinal column during embryonic development, with an incidence ranging from 1 to 10 per 1000 live births worldwide [1]. Among the subtypes of spina bifida, myelomeningocele (MMC) is the most severe form. MMC frequently presents with hydrocephalus, a condition characterized by an abnormal accumulation of cerebrospinal fluid (CSF) within the brain’s ventricles. Hydrocephalus develops in approximately 70–80% of newborns with MMC, typically within the first few weeks of life [1]. Effective management of hydrocephalus in these patients is critical to mitigating the risk of severe complications, including brainstem dysfunction resulting from worsening Chiari type II malformations, brain damage, or developmental delays caused by increased intracranial pressure [2, 3].

The standard treatment for hydrocephalus in MMC patients is the placement of a ventriculoperitoneal shunt (VPS), which diverts excess CSF from the ventricles to the peritoneal cavity, thereby relieving intracranial pressure [4]. Previous literature has established that VPS placement is effective in reducing ventricular volumes and stabilizing head circumference in children with hydrocephalus [5]. However, the impact of VPS on long-term changes in ventricle size and overall intracranial volume in MMC has not yet been well characterized. This knowledge gap is particularly relevant given the growing use of alternative surgical interventions, such as endoscopic third ventriculostomy (ETV), which can result in overall larger ventricle volumes when compared with VPS [6]. Understanding how ventricle size correlates with intracranial volume and clinical outcomes in MMC patients is crucial for optimizing treatment strategies and improving long-term neurodevelopmental outcomes.

This study uses MRI-based segmentation to quantify ventricular and intracranial volumes in children with MMC, comparing shunted and non-shunted patients to identify volumetric differences and potential implications for clinical decision-making.

## Methods

### Study design

This was a retrospective, single-center observational cohort study of patients who underwent MMC repair and were treated with and without VPS. The Institutional Review Board (IRB) approved the study under expedited review, and informed consent was waived due to the study’s retrospective design.

### Study population

**Fig. 1:**
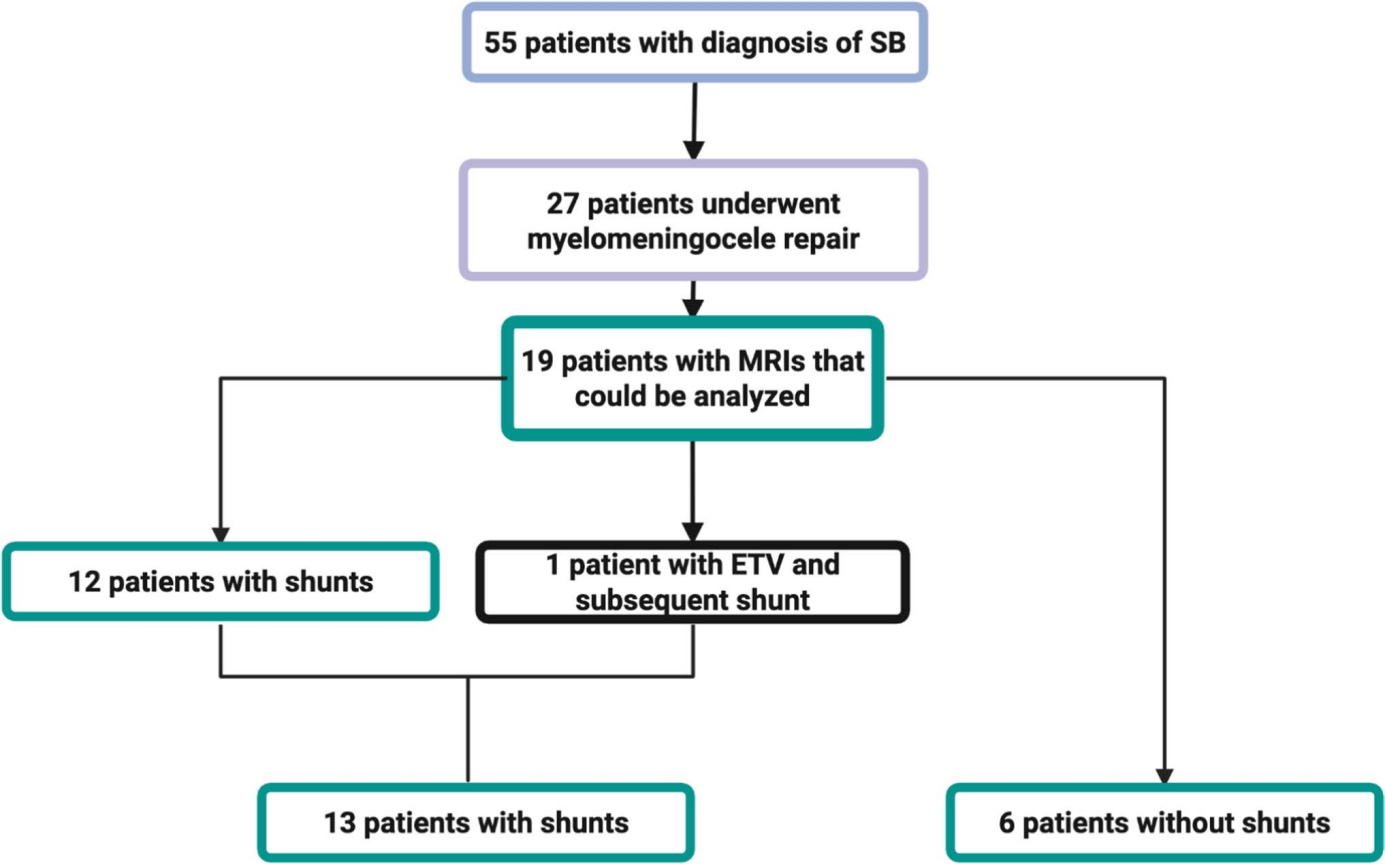
A flow diagram showing patient inclusion. Of 55 patients with spina bifida seen at our institution between 2013 and 2023, 28 were excluded because they did not undergo MMC repair. 8 patients were then excluded due to a lack of analyzable MRIs, leaving 19 patients in the final cohort – 6 patients without shunts and 13 with shunts, including one patient with an ETV that was subsequently converted to a shunt

Patients seen between November 2013 and November 2023 were identified from the electronic medical record (EMR) using SlicerDicer, an embedded data analysis tool [7]. Inclusion criteria were age 0 to 3 years old, a diagnosis of spina bifida with MMC repair (open or in utero), and the availability of at least one rapid-sequence MRI in the EMR (see “ MRI Protocols” for rapid-sequence details). Exclusion criteria included the presence of a significant brain malformation unrelated to spina bifida and the absence of a rapid-sequence MRI in the EMR. Medical records were screened by the senior author (H.S.) to confirm eligibility. Of the 55 patients initially identified, 28 did not undergo MMC repair. Of the remaining 27 patients, 19 had analyzable MRI scans and were included in the final cohort (Fig. 1).

### Data collection

After eligible patients were identified, demographic and clinical data were extracted from the EMR. Demographic information included gender, age, and gestational age. Clinical data collected included the type of MMC repair (open or fetal), head circumference, standard deviation (SD), the dates of any VP shunt placement or ETV procedure and repairs, type of shunt (fixed pressure or programmable), and any significant relevant comorbidities. MRI scans included in this study were performed as part of surveillance scans; shunt failure scans were not included. Outcomes measured included ventricle volume, intracranial volume, and ventricle-to-intracranial volume ratio (ventricle/ICV).

### Shunt types and categorization

Valve types included Codman Certas Plus**®**, Sophysa Polaris**®** SPV, and Delta**®**. Shunt settings were converted to pressure categories based on each valve’s pressure settings, as shown in Table 1 [8–10].

**Table 1:** Shunt valve types, settings, and pressures

Valve type	Valve setting (#, %)	Pressure range (mmH_2_O)	Pressure category
Codman Certas Plus®	1	25–45	Very low
	2	50–70	Low
	3	80–100	Low
	4	110–135	Medium
	5	145–180	High
	6	180–215	Very high
Sophysa Polaris® SPV	1	30	Very low
	2	70	Low
	3	110	Medium
	4	150	High
	5	200	Very high
Delta®	.5	10–30	Very low
	1	30–45	Low
	1.5	85–105	Medium
	2	145–170	High
	2.5	200–220	Very high

### MRI processing and analysis

Once patients were identified, accession numbers for all brain MRIs for each child were obtained from the EMR. To obtain the image files in DICOM format, a program developed by author XF was utilized (the program can be found here: https://www.carinaai.com/deidentifier.html). This program was used to query and retrieve MRI images from the Picture Archiving and Communication System (PACS).

**Fig. 2:**
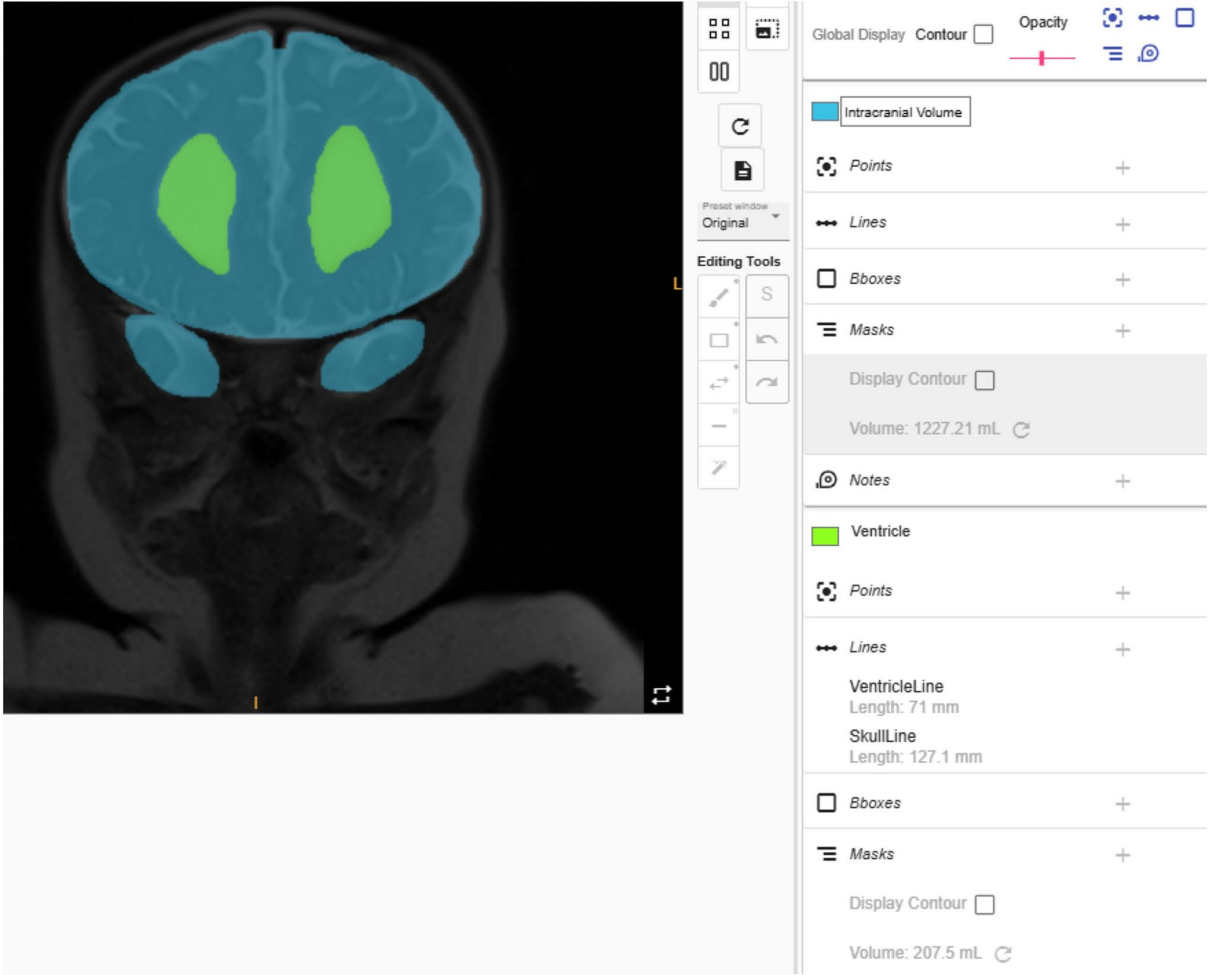
An example final MRI segmentation is shown (following initial automatic segmentation with subsequent manual correction). 2-dimensional intracranial masks (blue) and ventricle masks (green) are generated for each MRI slice, creating a 3-dimensional volume measurement. Editing tools are shown in the middle of the image, while ventricle and intracranial mask settings are shown in the right of the image

Images were deidentified and uploaded to a customized web-based program (https://www.carinaai.com/deidentifier.html) that allows for segmentation and calculation of ventricular volumes. The segmentation algorithm generated an initial mask of the ventricles and intracranial volumes, which were then manually corrected with a drawing tool (Fig. 2).

Ventricular and intracranial volumes were measured from rapid-sequence T2-weighted MRI scans using manual segmentation techniques. The ventricle/ICV ratio was calculated as the ratio of ventricular volume (mL) to total intracranial volume (mL). All volume measurements underwent quality control—initial masks were corrected by a single reviewer (author M.P.) and then reviewed by senior author H.S. to ensure accuracy. These final segmentations were used as the ground truth—an approach that aligns with prior manual segmentation studies [11–13]. Segmentation accuracy was confirmed using a simultaneously generated 3-dimensional model.

### MRI protocols

Ninety-nine MRIs were available from the included patients. Eight of these scans used a full brain MRI protocol and were excluded, leaving 91 rapid-sequence scans for use in this study. The institutional protocol for rapid-sequence scans was sagittal and coronal half Fourier single-shot turbo spin-echo (HASTE) and axial balanced steady state free precession line acquisition with under sampling (BLADE). All scans were T2-weighted with a 4 mm slice thickness and were performed on Siemens 1.5 or 3 Tesla systems.

### Primary objective

The primary objective of this study was to compare intracranial and ventricle volumes between shunted and non-shunted patients with MMC.

### Statistical analysis

MRI-derived intracranial and ventricular volumes were collected longitudinally, with multiple observations per subject. The primary outcome was the ventricle/ICV ratio, calculated at each imaging time point. Key covariates included age at MRI (modeled both continuously and categorically), gestational age at birth, head circumference z-scores, and shunt type. Age categories were defined to balance the number of scans across groups.

Analyses were conducted in R. Statistical significance is demonstrated in figures and tables such that * indicates *p* ≤ 0.05. Categorical variables (e.g., gender, prematurity) were compared using chi-square or Fisher’s exact tests; for shunted patients, shunt pressure categories were compared against continuous variables (ventricle and intracranial volumes) with ANOVA tests; continuous variables (gestational age, head circumference, ventricular and intracranial volumes, ventricle/ICV ratios) were compared using Mann–Whitney *U* tests; and the number of scans per patient was compared using the Wilcoxon rank-sum test.

Baseline volumetric characteristics were summarized with independent *t*-tests, with results reported as means and 95% confidence intervals. Given the size of the study population, effect sizes were calculated using Hedges’ *g* to quantify the magnitude of group differences, with large effect size *g* > 0.8. Longitudinal changes in ventricle/ICV ratios were analyzed using linear mixed-effects models with random intercepts for subjects and fixed effects for group, age, prematurity status, and head circumference. Group × age interaction terms were tested to assess differential developmental trajectories.

To strengthen inference beyond distributional assumptions, multiple sensitivity analyses were performed. Uncertainty was quantified using non-parametric bootstrap with 1000 re-samples, stratified by shunt status where applicable. Confidence intervals were percentile-based. Permutation tests were applied across developmental age categories (neonatal, infancy, toddlerhood). Robust regression within ≤ 50 days, adjusting for head circumference, was used to verify results and identify influential observations. Effect-size analyses were performed for early (≤ 50 days) and later (> 50 days) periods. Finally, joint modeling of ventricular and intracranial volumes was conducted to assess associations between volumetric growth patterns. Model diagnostics included inspection of residual distributions and identification of influential observations via Cook’s distance.

## Results

A total of 91 MRIs from 19 patients were analyzed. Of these, 13 (68.4%) had shunts and 6 (31.6%) did not (Table 2). Eight (42.1%) were female and 5 (26.3%) underwent fetal repair. Baseline characteristics did not differ significantly between groups, including sex (*p* = 1.000), gestational age (*p* = 0.681), prematurity < 35 weeks (*p* = 1.000), rates of cerebellar tonsillar herniation below the foramen magnum (*p* = 0.319), and initial head circumference z-scores (*p* = 0.060). The median number of scans per patient were similar (shunted 5.0 [IQR 4.0–6.0] versus non-shunted 4.5 [IQR 4.0–7.0]; *p* = 0.683). Among shunted patients, 4 (30.8%) had fixed-pressure valves and 9 (69.2%) had programmable valves. Programmable valve types included 7 (53.8%) Codman Certas Plus**®** and 1 (7.7%) Sophysa Polaris**®** SPV. All fixed-pressure valves were Delta**®** valves. One patient underwent ETV that was subsequently converted to VPS. No non-shunted patients received ETVs.

**Table 2:** Demographic information, *p*-value, and relevant statistical tests by shunt status

Variable	Shunted (*n* = 13)	No shunt (*n* = 6)	*p*	Test
Gender (%)				
F	6 (46.2)	2 (33.3)	1.000	Fisher’s Exact
M	7 (53.8)	4 (66.7)		
Gestational age at birth in days (median [IQR])	274.00 [255.00, 274.00]	265.00 [259.00, 273.25]	0.681	Mann Whitney U
Premature = < 35 weeks (#, %)				
N	10 (76.9)	4 (66.7)	1.000	Fisher’s Exact
Y	3 (25.0)	2 (33.3)		
Extension of cerebellar tonsils below foramen magnum (#, %)				
N	4 (30.8)	4 (66.7)	0.319	Fisher’s Exact
Y	9 (69.2)^**a**^	2 (33.3)		
Initial head Circ SD (median [IQR])	1.9 [0.41, 3.25]	1.98 [0.748, 2.94]	0.060	Mann Whitney U
Initial ventricle volume (mL) (median [IQR])	82.2 [52.4, 186.9]	34.2 [18.3, 59.4]	0.072	Mann Whitney U
Initial intracranial volume (mL) (median [IQR])	644.3 [527.7, 826.1]	403.5 [396.7, 447.1]	0.020*	Mann Whitney U
Vent/Intracranial ratio (median [IQR])	0.2 [0.047, 0.340]	0.1 [0.073, 0.147]	0.054	Wilcoxon rank-sum
Number of scans (median [IQR])	5.0 [4.0–6.0]	4.5 [4.0–7.0]	0.683	Wilcoxon rank-sum
Repair type (#, %)				
In Utero	4 (30.8)	1 (16.7)		
Open	9 (69.2)	5 (83.3)		
Shunt Type (#, %)				
Fixed Pressure	5 (38.5)			
Programmable	8 (61.5)			
Shunt timing (days)				
0–1	2			
2–7	4			
8–49	1			
50–149	5			
150–249	0			
250 +	1			
Shunt pressure category (#, %)				
Very Low	5 (9.4)			
Low	18 (34.0)			
Medium	22 (41.5)			
High	6 (11.3)			
Very High	2 (3.8)			
ETV (%)^b^				
No	12 (92.3)	6 (100.0)		
Yes	1 (7.7)	0 (0.0)		

^**a**^One patient with cerebellar tonsil herniation below the foramen magnum underwent posterior fossa decompression

^b^Patient initially had Endoscopic third ventriculostomy (ETV) then was subsequently shunted

### Intracranial volumes

**Fig. 3:**
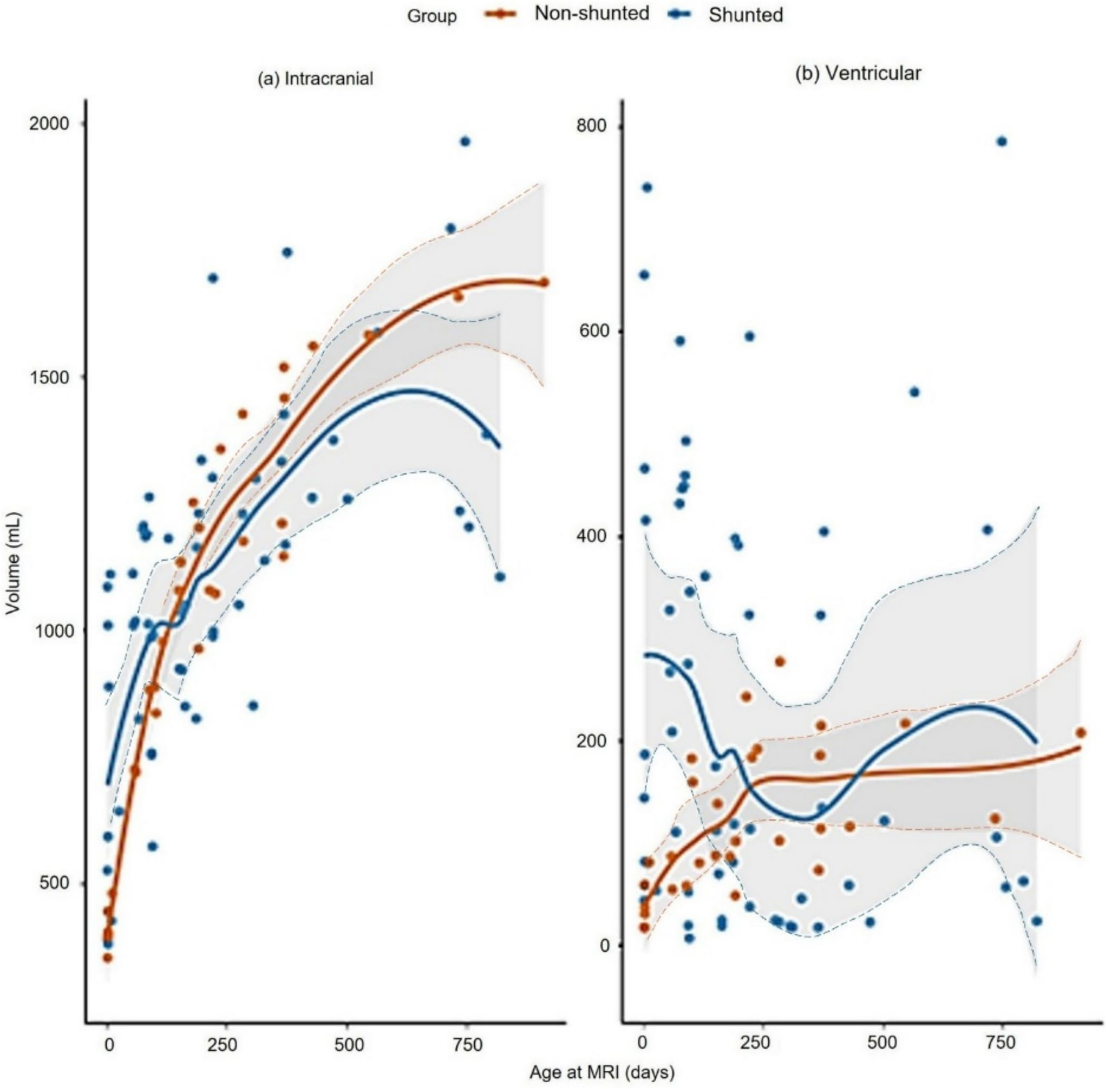
Scatterplots with locally estimated scatterplot smoothing trend lines (solid lines) and confidence intervals (dashed lines) showing intracranial (**a**) and ventricular (**b**) volumes by age at MRI (days). Shunted patients demonstrated significantly larger intracranial volumes at baseline but not beyond 100 days of age. Ventricular volumes tended to be higher in shunted patients earlier and higher ventricular volumes in non-shunted patients later, though these differences were not statistically significant

Intracranial volume increased with age in both cohorts. Median intracranial volumes by valve setting were as follows: 1967 mL [IQR 852.6–1967] very low, 1206.5 mL [1057–1616.5] low, 1248.5 mL [1051.5–1374.5] medium, 1006.3 mL [943.7–1083.6] high, and 1139.5 [1113.5–1165.4] very high (F(4,48) = 0.90, *p* = 0.474). At baseline, intracranial volumes were higher in shunted patients (median 644.3 mL [527.7–826.1] vs. 403.5 mL [396.7–447.1]; *p* = 0.020). After 100 days, median intracranial volumes were comparable (*p* = 0.707). At 150 days and over 400 days, non-shunted patients had higher median intracranial volumes (1253.5 vs. 1234.8 mL and 1621.8 vs. 1320.0 mL, respectively), though these differences were not significant (*p* = 0.432 and *p* = 0.179) (Fig. 3).

### Ventricular volumes

At baseline, shunted patients had larger ventricular volumes, though not significantly so (median 82.2 mL [IQR 52.4–186.9] vs. 34.2 mL [18.3–59.8]; *p* = 0.072). Median ventricular volumes by valve setting were as follows: 787 mL [IQR 186.9–787] very low, 225.3 mL [47.6–566.7] low, 114 [43.3–385] medium, 252.7 mL [112.9–409.7] high, and 552.7 mL [501.2–604.1] very high (F(4, 48) = 1.35, *p* = 0.266). Figure 3 shows that after 100, 150, and over 400 days, ventricular volumes tended to be higher in the non-shunted group (131.4 vs. 106.0 mL; 138.7 vs. 75.9 mL; 166.1 vs. 84.5 mL), but these differences were not statistically significant (*p* = 0.100, 0.200 and 0.374, respectively).

### Ventricular/intracranial volume ratio

**Fig. 4:**
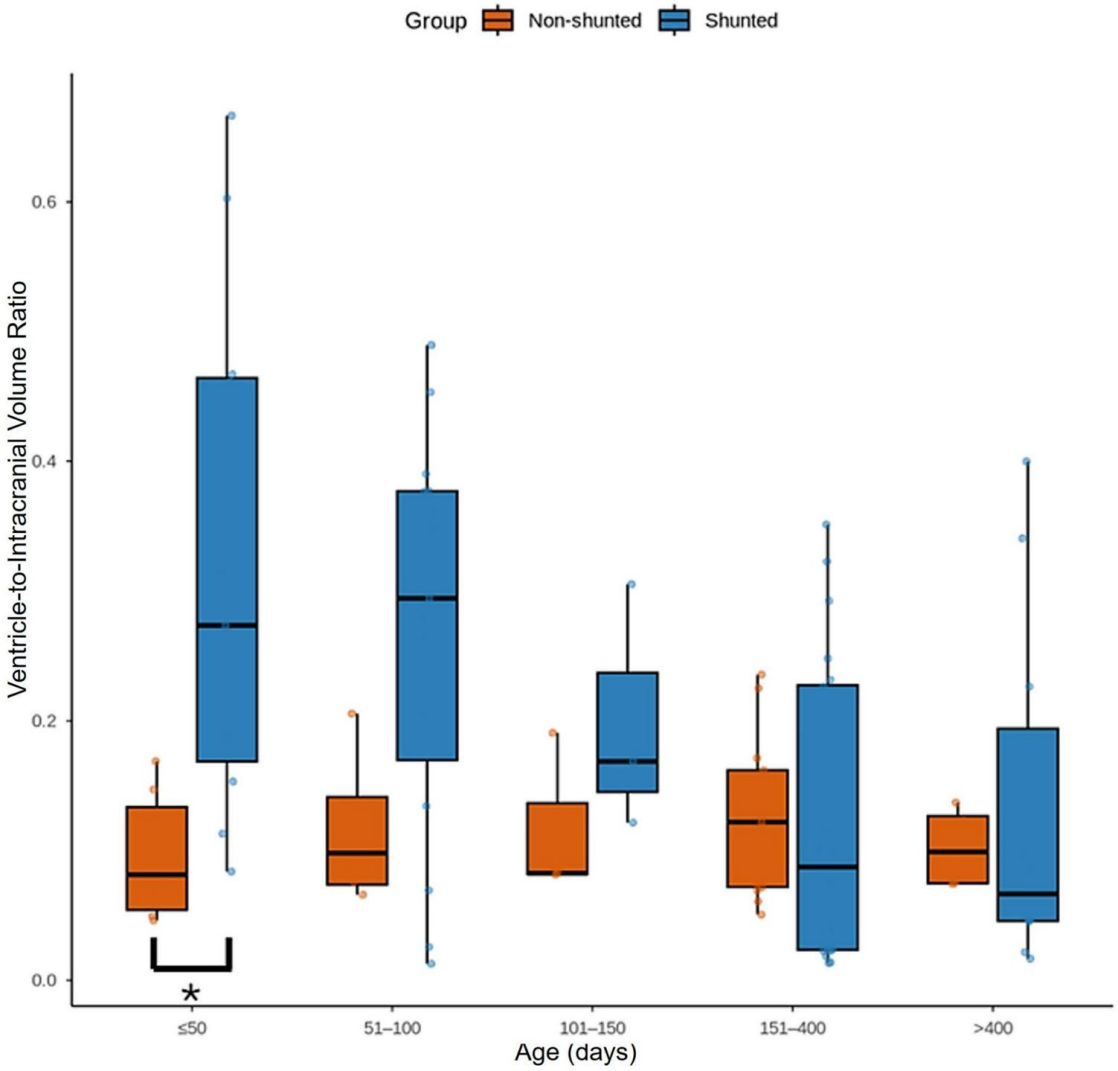
A box-and-whisker plot showing the distribution of ventricle/ICV ratios in shunted (orange) and non-shunted (blue) patients, grouped by age (less than 50 days old, 51–100, 101–150, 151–400, and over 400 days old). Ventricle/ICV ratios were significantly higher in the first 50 days

At baseline, the ventricle/ICV ratios were higher in shunted versus non-shunted patients (median 0.2 [*n* = 13] vs. 0.1 [*n* = 6]; *p* = 0.174). Within the first 50 days, ratios were significantly higher in shunted patients (*p* = 0.026; *n* = 17 observations), but this association attenuated after adjusting for head circumference z-score and prematurity (*p* = 0.144; *n* = 17). Beyond 50 days, no significant differences were observed in any age window examined: 51–100 days (*p* = 0.142; *n* = 19: 15 shunted, 4 non-shunted), 101–150 days (*p* = 0.372; *n* = 6: 3 shunted, 3 non-shunted), 151–400 days (*p* = 0.898; *n* = 33: 20 shunted, 13 non-shunted), > 400 days (*p* = 0.762; *n* = 14: 10 shunted, 4 non-shunted), and overall > 50 days (*p* = 0.410; *n* = 72: 48 shunted, 24 non-shunted) (Fig. 4).

### Ventricle/ICV ratio analyses across developmental stages

Bootstrap resampling confirmed significantly higher ventricle/ICV ratios in shunted patients within the first 50 days of life (difference = 0.192, 95% CI [0.033–0.402]). Bootstrap confidence intervals and *p*-values are based on 1000 re-samples; repeating with 5000 re-samples did not change inference. Permutation testing showed significant differences in the neonatal period (≤ 28 days; *p* = 0.013; *n* = 6 non-shunted, 11 shunted), but not in infancy (29–365 days; *p* = 0.095; *n* = 17 non-shunted, 35 shunted) or toddlerhood (366–1028 days; *p* = 0.518; *n* = 7 non-shunted, 13 shunted) (Table 3). Robust regression within ≤ 50 days, adjusting for head circumference, supported these findings. Five outliers were identified in this analysis; however, models with down-weighting of these scans and sensitivity checks excluding them from analysis produced similar results. Effect size analyses demonstrated a large magnitude difference in the early (≤ 50-day) period (Hedges’ *g* = 1.268) that diminished later (*g* = 0.452). Joint modeling suggested a positive but nonsignificant association between ventricular and intracranial volume growth (slope = 0.089, *p* = 0.065).

**Table 3:** Ventricle/ICV ratio by developmental stage and shunt status and permutation testing *p*-values

Developmental stage (age)	Ventricle/ICV ratio (median [IQR])		***p***
	Shunted (***n*** = 13)	No shunt (***n*** = 6)	
Neonatal (≤ 28 days)	0.27 [0.17, 0.46]	0.08 [0.05, 0.13]	*p* = 0.013*
Infancy (29–365 days)	0.13 [0.03, 0.31]	0.08 [0.07, 0.017]	*p* = 0.095
Toddlerhood (366–1028 days)	0.10 [0.05, 0.23]	0.12 [0.08, 0.14]	*p* = 0.518

## Discussion

This single-center study evaluated longitudinal intracranial and ventricular volumes in shunted and non-shunted children with myelomeningocele (MMC) using automated MRI segmentation with manual correction. Baseline demographics were comparable between groups. Shunted patients demonstrated larger, though not statistically significant, ventricular volumes at baseline and significantly larger intracranial volumes within the first 100 days of life. Ventricle-to-intracranial volume (ventricle/ICV) ratios were significantly higher in shunted patients during the first 50 days; however, this difference was attenuated after adjustment for head circumference and prematurity. Sensitivity analyses—including bootstrap confidence intervals, robust regression, and effect size estimation (Hedges’ *g*)—consistently supported higher ventricle/ICV ratios in shunted patients during the neonatal period, with additional confirmation through permutation testing. Importantly, after 50 days of life, no significant differences in intracranial or ventricular volumes were observed between the two groups. These findings suggest that early ventricular enlargement in shunted patients could potentially be a reproducible phenomenon, but that long-term ventricle/ICV ratios trajectories may converge with those of non-shunted patients.

These results support previous literature showing similar ventricular enlargement between shunted and non-shunted individuals [14–16]. In this cohort, more than half (53.8%) of shunted patients underwent VPS placement within the first 50 days of life, consistent with the trend of shunt placement early in life in MMC-associated hydrocephalus [17–19]. Taken together, these results raise the possibility that ventricular and intracranial volumes in shunted patients may follow trajectories similar to those of their non-shunted counterparts over time, though causal interpretation is limited by the study’s retrospective design.

The use of the ventricle/ICV ratio was particularly informative, revealing group differences that were not apparent when only ventricular volumes were examined. Ventricle-to-brain and ventricle-to-intracranial volume ratios have been applied in other pediatric hydrocephalus contexts, including posthemorrhagic hydrocephalus and posterior fossa tumors [20, 21], but, to our knowledge, not previously in MMC. This study, therefore, introduces ventricle/ICV ratios as a useful marker for comparison of MMC patients.

The primary aim of this study was to clarify whether ventricle/ICV ratios differ significantly between shunted and non-shunted MMC patients, given persistent uncertainty regarding the degree of ventricular enlargement that can be safely tolerated without surgical intervention. Previous investigations employing diffusion tensor imaging (DTI) in patients with MMC and hydrocephalus have not demonstrated a consistent association between ventricular size and white matter tract integrity [22–25]. Indeed, several studies have reported that greater ventricular size is correlated with higher fractional anisotropy—an established marker of white matter tract integrity [22–24]—as well as with more favorable clinical outcomes [23]. Despite these observations, consensus has not been reached. Our findings contribute to this body of literature by demonstrating that, when normalized to intracranial volume, ventricular size in shunted and non-shunted patients do not differ significantly at later time points. Further study is required to determine whether these findings translate to a lack of significant clinical difference between shunted and non-shunted MMC patients.

## Limitations

This study has several strengths, most notably detailed manual segmentation of rapid-sequence MRI scans, enabling precise volumetric quantification. However, the study is limited in several ways. Shunted patients may have had more severe presentations, introducing confounding by indication. In addition, the study’s retrospective design precludes the establishment of causal relationships. Use of fast MRI protocols may have impacted segmentation accuracy. The small sample size from a single center increases the probability of type II error and limits generalizability. The use of multiple MRI scans per patient enabled longitudinal analysis of volumes but introduced potential bias into the analysis that likely persisted despite statistical adjustment. Manual segmentation, although carefully reviewed, is subject to interobserver variability. The use of shunt settings from clinical notes to categorize shunts rather than true valve pressures introduces potential inaccuracies into the shunt valve setting analyses. Finally, the volumes analyzed in this study (ventricular, intracranial, and ventricular-to-intracranial volume ratios) have empiric clinical value but do not directly translate to functional outcomes, underscoring the need to incorporate neurocognitive measures in future work.

## Future directions

Future work should apply these segmentation methods to a larger cohort of patients with MMC to establish more robust trends in the effect of VPS placement on ventricular and intracranial volumes in the context of this pathology. In addition, prospective studies may be used to explore potential relationships between shunting and ventricular sizes in MMC patients over time. Integration of functional outcomes would further strengthen the clinical relevance of these volumetric findings.

## Conclusion

Children with MMC who underwent ventriculoperitoneal shunting exhibited significantly higher intracranial volumes and ventricle/ICV ratios during the neonatal period (< 50 days). However, these differences were not observed beyond this early window. These findings suggest potential convergence of ventricular dynamics between shunted and non-shunted patients over time.. Furthermore, this study also showed that non-shunted patients demonstrated comparable long-term intracranial volume growth trajectories compared to their shunted counterparts.

## Data Availability

No public datasets were generated or analyzed during the current study in order to protect patient privacy.

## References

[1] Saarinen O, Piironen S, Pokka T et al (2024) To shunt or not to shunt when closing myelomeningocele? A systematic review and meta-analysis of simultaneous versus delayed ventriculoperitoneal shunt placement in neonates undergoing myelomeningocele closure. 10.3171/2024.5.PEDS23600 10.3171/2024.5.PEDS23600 39126714

[2] Hashimoto H, Irizato N, Takemoto O, Chiba Y (2024) Intracranial volumetric evaluation in postnatally repaired myelomeningocele infants. Childs Nerv Syst 40(9):2851–2858. 10.1007/s00381-024-06444-2 38714605 10.1007/s00381-024-06444-2 PMC11322201

[3] Blount JP, Maleknia P, Hopson BD, Rocque BG, Oakes WJ (2021) Hydrocephalus in spina bifida. Neurol India 69(Suppl 2):S367. 10.4103/0028-3886.332247 35102990 10.4103/0028-3886.332247

[4] Lu VM, Shimony N, Jallo GI, Niazi TN (2024) Infant hydrocephalus. Pediatr Rev 45(8):450–460. 10.1542/pir.2023-006318 39085190 10.1542/pir.2023-006318

[5] Dewan MC, Lim J, Gannon SR et al (2018) Comparison of hydrocephalus metrics between infants successfully treated with endoscopic third ventriculostomy with choroid plexus cauterization and those treated with a ventriculoperitoneal shunt: a multicenter matched-cohort analysis. 10.3171/2017.10.PEDS17421 10.3171/2017.10.PEDS17421 29393809

[6] Azab WA, Mijalcic RM, Nakhi SB, Mohammad MH (2016) Ventricular volume and neurocognitive outcome after endoscopic third ventriculostomy: is shunting a better option? A review. Childs Nerv Syst 32(5):775–780. 10.1007/s00381-016-3032-3 26861009 10.1007/s00381-016-3032-3

[7] Saini V, Jaber T, Como JD, Lejeune K, Bhanot N (2021) Exploring ‘Slicer Dicer’, an extraction tool in EPIC, for clinical and epidemiological analysis. Open Forum Infectious Diseases 8(Supplement_1):S414–S415

[8] (n.d.) CERTAS^TM^ Plus programmable valves. https://products.integralife.com/file/general/1549983654.pdf. Accessed 4 Mar 2026

[9] POLARIS® VALVE: Instructions for Use. https://www.sophysa.us/wp-content/uploads/2023/11/Polaris%C2%AE-Instructions-for-Use.pdf. Accessed 4 Mar 2026

[10] Scholz R, Lemcke J, Meier U, Stengel D (2018) Efficacy and safety of programmable compared with fixed anti-siphon devices for treating idiopathic normal-pressure hydrocephalus (iNPH) in adults – SYGRAVA: study protocol for a randomized trial. Trials 19:566. 10.1186/s13063-018-2951-6 30333067 10.1186/s13063-018-2951-6 PMC6192316

[11] Breakey W, Knoops PGM, Borghi A et al (2017) Intracranial volume measurement: a systematic review and comparison of different techniques. J Craniofac Surg 28(7):1746. 10.1097/SCS.0000000000003929 28962091 10.1097/SCS.0000000000003929

[12] Malone IB, Leung KK, Clegg S et al (2015) Accurate automatic estimation of total intracranial volume: a nuisance variable with less nuisance. Neuroimage 104:366–372. 10.1016/j.neuroimage.2014.09.034 25255942 10.1016/j.neuroimage.2014.09.034 PMC4265726

[13] Ambarki K, Lindqvist T, Wåhlin A et al (2012) Evaluation of automatic measurement of the intracranial volume based on quantitative MR imaging. AJNR Am J Neuroradiol 33(10):1951–1956. 10.3174/ajnr.A3067 22555574 10.3174/ajnr.A3067 PMC7964602

[14] Warf B, Ondoma S, Kulkarni A et al (2009) Neurocognitive outcome and ventricular volume in children with myelomeningocele treated for hydrocephalus in Uganda. 10.3171/2009.7.PEDS09136 10.3171/2009.7.PEDS09136 19951045

[15] Warf BC, Weber DS, Day EL et al (2023) Endoscopic third ventriculostomy with choroid plexus cauterization: predictors of long-term success and comparison with shunt placement for primary treatment of infant hydrocephalus. 10.3171/2023.4.PEDS2310 10.3171/2023.4.PEDS2310 37178026

[16] Nikas DC, Post AF, Choudhri AF, Mazzola CA, Mitchell L, Flannery AM (2014) Pediatric hydrocephalus: systematic literature review and evidence-based guidelines. Part 10: change in ventricle size as a measurement of effective treatment of hydrocephalus. 10.3171/2014.7.PEDS14330 10.3171/2014.7.PEDS14330 25988786

[17] McCarthy DJ, Sheinberg DL, Luther E, McCrea HJ (2019) Myelomeningocele-associated hydrocephalus: nationwide analysis and systematic review. 10.3171/2019.7.FOCUS19469 10.3171/2019.7.FOCUS19469 31574479

[18] İştemen İ, Arslan A, Olguner SK, Açik V, Ökten Aİ, Babaoğlan M (2021) Shunt timing in meningomyelocele and clinical results: analysis of 80 cases. Childs Nerv Syst 37(1):107–113. 10.1007/s00381-020-04786-1 32632579 10.1007/s00381-020-04786-1

[19] Ozgural O, Kahilogullari G, Dogan I et al (2019) Timing of shunt insertion in children with neural tube defects and hydrocephalus: clinical study. Turkish Neurosurg. 10.5137/1019-5149.JTN.26588-19.1 10.5137/1019-5149.JTN.26588-19.1 32153000

[20] Grimm F, Edl F, Gugel I, Kerscher SR, Bender B, Schuhmann MU (2020) Automatic volumetry of cerebrospinal fluid and brain volume in severe paediatric hydrocephalus, implementation and clinical course after intervention. Acta Neurochir 162(1):23–30. 10.1007/s00701-019-04143-5 31768752 10.1007/s00701-019-04143-5

[21] Wilhelmy F, Güresir E, Wach J et al (2025) Volumetric predictors for shunt-dependency in pediatric posterior fossa tumors. Sci Rep 15:20235. 10.1038/s41598-025-06825-w 40542153 10.1038/s41598-025-06825-w PMC12181244

[22] Williams VJ, Juranek J, Stuebing KK et al (2015) Postshunt lateral ventricular volume, white matter integrity, and intellectual outcomes in spina bifida and hydrocephalus. 10.3171/2014.10.PEDS13644 10.3171/2014.10.PEDS13644 25634821

[23] Tan K, Meiri A, Mowrey WB et al (2018) Diffusion tensor imaging and ventricle volume quantification in patients with chronic shunt-treated hydrocephalus: a matched case-control study. 10.3171/2017.6.JNS162784 10.3171/2017.6.JNS162784 29350598

[24] Ragguett RM, Eagleson R, de Ribaupierre S (2024) Association between altered white matter networks and post operative ventricle volume in shunt-treated pediatric hydrocephalus. Brain Res Bull 206:110847. 10.1016/j.brainresbull.2023.110847 38103800 10.1016/j.brainresbull.2023.110847

[25] Kulkarni AV, Donnelly R, Mabbott DJ, Widjaja E (2015) Relationship between ventricular size, white matter injury, and neurocognition in children with stable, treated hydrocephalus. 10.3171/2015.1.PEDS14597 10.3171/2015.1.PEDS14597 26046689

